# Maternal Use of Integrase Strand Transfer Inhibitors During Pregnancy and Infant Neurodevelopment

**DOI:** 10.1001/jamanetworkopen.2025.45652

**Published:** 2025-11-26

**Authors:** Paige L. Williams, Martha Boahene, Lisa E. Mash, Ellen G. Chadwick, Yanling Huo, Stephen A. Spector, Janna Oetting, Patricia A. Garvie, Renee Smith

**Affiliations:** 1 Department of Biostatistics, Harvard T. H. Chan School of Public Health, Boston, Massachusetts; 2 Center for Biostatistics in AIDS Research, Harvard T. H. Chan School of Public Health, Boston, Massachusetts; 3 Department of Psychiatry, Northwestern University Feinberg School of Medicine, Chicago, Illinois; 4 Division of Infectious Diseases, Department of Pediatrics, Northwestern University Feinberg School of Medicine, Chicago, Illinois; 5 Division of Infectious Diseases, Department of Pediatrics, University of California, San Diego, and Rady Children’s Hospital, San Diego; 6 Department of Communication Sciences and Disorders, Louisiana State University, Baton Rouge; 7 Children’s Diagnostic & Treatment Center, Ft Lauderdale, Florida; 8 Department of Pediatrics, University of Illinois at Chicago, Chicago

## Abstract

**Importance:**

Current perinatal HIV treatment guidelines recommend inclusion of integrase strand transfer inhibitors (INSTIs), but few studies have evaluated the safety of INSTIs for infant neurodevelopment.

**Objective:**

To compare the neurodevelopment of infants born between 2012 and 2023 with in utero exposure to INSTI-containing antiretroviral treatment (ART) regimens with that of infants exposed to protease inhibitor (PI)–containing or nonnucleoside reverse transcriptase inhibitor (NNRTI)–containing regimens.

**Design, Setting, and Participants:**

This prospective cohort study was conducted in 22 US-based academic research centers among women with HIV and their uninfected infants born between January 1, 2012, and December 31, 2023, and exposed in utero to INSTI-based, PI-based, or NNRTI-based combination ART regimens. The children underwent neurodevelopmental assessments at 1 year of age. Data were analyzed between January 13 and September 5, 2025.

**Exposure:**

Initial maternal ART regimen during pregnancy.

**Main Outcomes and Measures:**

Neuropsychologists administered the Bayley Scales of Infant and Toddler Development–3rd Edition (Bayley-III) to children at 1 year of age. Mean differences by drug class were calculated for Bayley-III cognitive, language, and motor development domain scores, adjusting for confounders. Stratified analyses were conducted by timing of maternal ART initiation.

**Results:**

A total of 1006 infants born between 2012 and 2023 with a Bayley-III assessment at 1 year of age were included. The mean (SD) maternal age at delivery was 30.3 (6.0) years; 663 mothers (65.9%) had an annual income of less than $20 000; and 125 (12.4%) had perinatally acquired HIV. Of the 1006 infants, 306 were exposed in utero to an initial INSTI-based maternal ART regimen, 473 to a PI-based regimen, and 227 to an NNRTI-based regimen; 531 (52.8%) were exposed from conception. Overall, mean (SD) scores for cognitive (101.7 [14.2]), language (95.5 [13.8]), and motor development (96.4 [13.2]) domains were near reference standards (100 [15]). There was no difference in Bayley-III cognitive, language, or motor development domain scores between those exposed to INSTI-based vs PI-based regimens. However, INSTI-exposed infants had slightly lower mean scores than infants exposed to NNRTI-based regimens for cognitive (adjusted mean difference, −2.94; 95% CI, −5.47 to −0.41), language (adjusted mean difference, −1.96; 95% CI, −4.50 to 0.57), and motor development (adjusted mean difference, −1.60; 95% CI, −4.15 to 0.94) domains, with larger differences among infants whose mothers initiated ART during pregnancy.

**Conclusions and Relevance:**

In this cohort study, infants exposed in utero to INSTI-based ART regimens had mean neurodevelopmental scores similar to those for infants exposed to PI-based regimens but lower than those for infants exposed to NNRTI-based regimens, particularly when their mothers initiated ART after conception. Notably, adjusted mean cognitive domain scores for INSTI-exposed infants were close to the expected population mean of 100, while NNRTI-exposed infants scored 4 to 5 points above the population mean. These findings support continued inclusion of INSTIs in first-line regimens for pregnant women with HIV.

## Key Points

**Question:**

Do integrase strand transfer inhibitors (INSTIs) used as part of antiretroviral treatment (ART) by pregnant women with HIV have an adverse association with their infant’s neurodevelopment?

**Findings:**

In this cohort study of 1006 children aged 1 year born to women with HIV, assessments of cognition, language, and motor development yielded no clinically meaningful differences between those exposed in utero to INSTI-based ART regimens and other types of ART regimens. Mean scores were close to the standard population mean of 100 within each ART exposure group.

**Meaning:**

These findings support current recommendations to include INSTI-based regimens as part of first-line treatment for pregnant women with HIV.

## Teaser

This cohort study evaluates neurodevelopmental outcomes among infants exposed in utero to antiretroviral therapy consisting of integrase strand inhibitor–based regimens compared with protease inhibitor– or nonnucleoside reverse transcriptase inhibitor–based regimens.

## Introduction

Globally, the number of children who are perinatally exposed to HIV but are uninfected continues to grow. Each year, more than 1 million women with HIV become pregnant, yet effective antiretroviral therapy (ART) during pregnancy has reduced vertical transmission to less than 1% in middle- and high-income countries.^1^ The expanding population of children who are HIV exposed but uninfected and changes in ART regimens used in pregnancy underscore a critical need to assess the safety of antiretroviral medications that have helped achieve this dramatic reduction in perinatal HIV transmission.^2,3^

While adverse birth outcomes have been well studied, neurodevelopmental effects among children who are exposed to HIV but are not infected remain a concern.^4,5^ Early in the HIV epidemic, when ART was not universally available, Williams et al^6^ found no difference in early development between HIV-uninfected infants who were or were not exposed to any ART in utero. Subsequent studies have generally been reassuring.^7,8,9^ For example, Sirois et al^7^ observed no differences in Bayley Scales of Infant and Toddler Development–3rd Edition (Bayley-III) scores between 1-year old HIV-uninfected children who had been exposed to combination ART and those exposed to mono-ART or dual-ART. However, exposure to certain antiretroviral drugs such as nelfinavir mesylate and atazanavir sulfate was associated with lower mean cognitive and language scores, respectively, as later confirmed by Caniglia et al.^10^ A 2022 meta-analysis^11^ reported poorer expressive language and gross motor function in HIV-uninfected children who had been exposed to HIV compared with uninfected infants who were not exposed , but found little evidence of associations with specific maternal ART regimens.

Past studies evaluating neurodevelopment in children who have been exposed to HIV but were not infected have focused on intrauterine exposure to nucleoside reverse-transcriptase inhibitors (NRTIs), nonnucleoside reverse-transcriptase inhibitors (NNRTIs), and protease inhibitors (PIs), either as overall drug classes or for specific drugs within these classes.^4,7,8,11,12^ Current World Health Organization and US guidelines recommend use of integrase strand transfer inhibitors (INSTIs), including dolutegravir (approved in 2013) or raltegravir potassium (approved 2007), for treatment of HIV infection.^13^ Other INSTIs used for maternal treatment include elvitegravir (2014) and bictegravir (2018). Second-generation INSTIs generally offer improved efficacy and higher resistance barriers than other drug classes,^14^ and some studies suggest lower risk of adverse perinatal outcomes with maternal use of INSTI-based regimens.^15,16^ Dolutegravir has been recommended as a preferred first-line agent in pregnancy since 2018 due to potent viral suppression, once-daily dosing, and lower prevalence of adverse effects.^1^ However, limited data exist on associations between in utero INSTI exposure and infant neurodevelopment. The aim of this study was to evaluate neurodevelopmental outcomes among infants exposed to but uninfected with HIV who were exposed in utero to INSTI-based regimens compared with NNRTI- or PI-based regimens using data from the Surveillance Monitoring for ART Toxicities (SMARTT) study conducted within the Pediatric HIV/AIDS Cohort Study (PHACS) network.

## Methods

### Study Population

We used data collected from pregnant women with HIV and their liveborn uninfected infants enrolled in SMARTT, a prospective cohort study conducted at 22 US sites (including Puerto Rico) beginning in 2007.^17,18,19^ Women with HIV were enrolled during gestation or within 1 week after delivery; infants were enrolled prenatally or shortly after birth and confirmed to be uninfected with HIV according to standard guidelines. Data on maternal health and ART exposures during pregnancy were collected by medical record abstraction and interview. Follow-up visits occurred annually through 5 years of age and then biannually until 17 years of age. To focus on the period of wider INSTI availability, we included infants born between January 1, 2012, and December 31, 2023, with a valid Bayley-III and/or MacArthur Bates Communication Development Inventory (MCDI) Words and Gestures assessments at the 1-year visit. We excluded infants whose mothers received regimens with more than 2 antiretroviral classes and those not receiving combination ART.

The SMARTT study was approved by Institutional Review Boards at participating sites and at Harvard T. H. Chan School of Public Health. Pregnant women provided written informed consent for themselves and their infants. We followed the Strengthening the Reporting of Observational Studies in Epidemiology (STROBE) reporting guideline.

### Neurodevelopmental Outcomes

At the 1-year study visit (when infants were aged 9-16 months), neuropsychologists administered the Bayley-III and MCDI assessments according to standardized procedures. The Bayley-III domains of cognition, language, and motor development were administered via direct infant interaction, yielding age-standardized scores with a population mean of 100 (SD, 15); these were considered the primary outcomes.^7,10,20^

As secondary outcomes, we considered MCDI assessments of language functioning. The MCDI evaluates parent-reported language functioning and yields age- and sex-adjusted percentile scores in 4 domains: A-E total gestures (measuring 5 nonverbal communication labeled A through E, such as pointing, reaching, or nodding), phrases understood, vocabulary comprehension, and word production.^21,22^ We considered low language functioning within each domain as an MCDI score at or less than the 10th percentile. All test results were reviewed for validity by neuropsychologists (J.O. and R.S.). While the MCDI assessment was available in English and Spanish, the Bayley-III was administered only in English. Both the Bayley-III and the MCDI are standard validated assessments widely used in the general population to assess early development and screen for developmental delays.

### Comparisons of In Utero ART Exposures

The primary exposure of interest was the initial maternal ART regimen in pregnancy, categorized as INSTI, PI, or NNRTI based; all regimens compared included a dual NRTI backbone. To best inform clinical practice, analyses were also stratified by timing of ART initiation (preconception vs during pregnancy).

### Covariates

Potential confounders were identified via a directed acyclic graph based on prior literature on child neurodevelopment and expected associations with maternal ART regimens. Confounders included maternal educational attainment, household income, geographic region, birth year, maternal perinatal HIV status, maternal mental health diagnosis, age at delivery, and first trimester substance use (alcohol, tobacco, marijuana, and/or illicit drugs, modeled separately). Other covariates summarized to describe the study population were primary language, change of caregiver, housing situation, maternal employment status, and infant sex. Race was self-reported by participants as American Indian or Alaska Native, Asian, Black or African American, Native Hawaiian or Other Pacific Islander, White, multiracial, or do not wish to report. Ethnicity was self-reported as Hispanic, non-Hispanic, more than one ethnicity, or do not wish to report. Race and ethnicity were collected according to National Institutes of Health human subjects requirements and summarized to describe the population. Variables on the causal pathway (occurring after the exposure and expected to be affected by the exposure [INSTIs] and also known or expected to be related to the outcome [neurodevelopment]), such as preterm delivery, early intervention services, and maternal CD4 count and HIV viral load (VL) in pregnancy, were described by exposure status but excluded from primary models.

### Statistical Analysis

Study population characteristics were summarized by maternal ART regimen and timing of ART initiation. Mean Bayley-III and MCDI scores, as well as proportions with low scores (>1.5 SDs below population mean for Bayley-III and ≤10th percentile for MCDI), were reported overall and by ART regimen. In our primary analyses, we calculated doubly robust estimators that combined inverse probability of treatment weighting with linear regression of outcomes to estimate the causal effects of infant exposure to an INSTI-based regimen compared with PI-based or NNRTI-based regimens.^23,24^ For the Bayley-III outcomes, we calculated mean differences in domain scores; for the MCDI, we estimated risk differences in the percentage with low MCDI scores.^23,24^ Separate pairwise comparisons were made to compare INSTI-based regimens with NNRTI- and PI-based regimens. Although missing data were minimal, missing indicators were used for certain covariates (income, educational attainment, substance use), since those participants who chose not to report these data were considered informative as a category.

We conducted several sensitivity analyses to assess the robustness of findings for the primary Bayley-III outcomes: (1) accounting for correlation of outcomes among siblings using generalized estimating equation linear regression models with an exchangeable correlation structure; (2) restricted to infants whose mothers continued the same regimen category throughout pregnancy; (3) accounting for maternal health based on the earliest available measures of CD4 level and VL during pregnancy; and (4) evaluating differences by timing of ART initiation by trimester within each regimen category. Given interest in specific INSTI medications, we conducted exploratory analyses to examine associations of individual antiretroviral medications with Bayley-III scores using a hierarchical linear model to simultaneously account for all antiretroviral medications used during pregnancy, as previously described.^25^ The hierarchical linear model approach includes fixed effects for each drug class and a random effect for each drug within its class, yielding individual antiretroviral estimates as additive deviations from drug class mean. Antiretroviral medications with low exposure borrow information from other drugs within the same drug class, under the assumption of a common mechanism of action.^26,27,28^ A multivariable model with fixed effects for all individual antiretroviral medications was also fit for comparison. Both approaches adjusted for the same covariates included in primary models.

Data were analyzed between January 13 and September 5, 2025. All analyses were conducted using SAS, version 9.4 (SAS Institute Inc). Two-sided *P* < .05 indicated statistical significance.

## Results

### Study Population and Participant Characteristics

**Figure 1.**
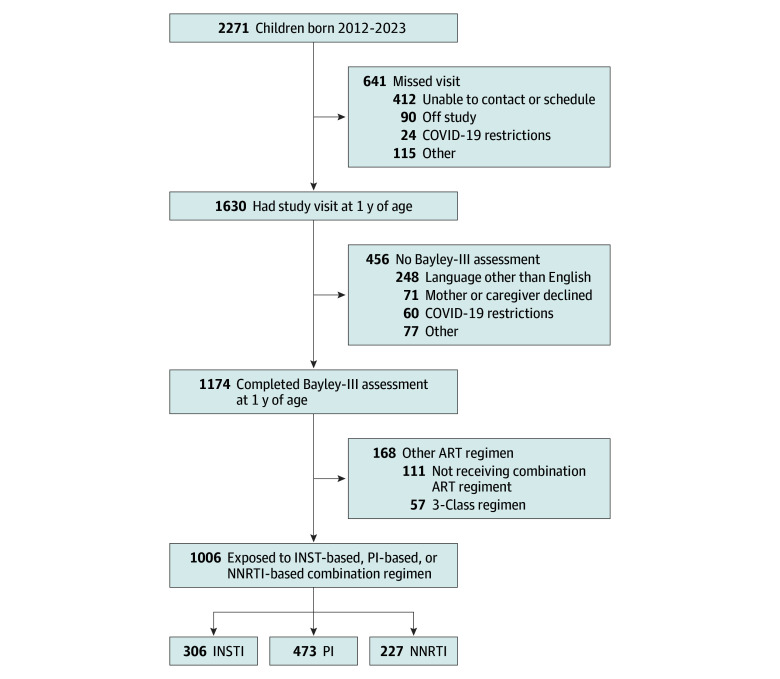
Surveillance Monitoring for ART (Antiretroviral Therapy) Toxicities Study Population for Bayley Scales of Infant and Toddler Development–3rd Edition (Bayley-III) Neurodevelopmental Analysis Derivation of study population for the analysis of scores from the Bayley-III neurodevelopmental assessment, showing the number included by initial maternal ART regimen. INSTI indicates integrase strand transfer inhibitor; NNRTI, nonnucleoside reverse transcriptase inhibitor; and PI, protease inhibitor.

Among 2271 infants born between 2012 and 2023, 1630 attended their 1-year visit and 1174 completed the Bayley-III assessment (Figure 1). Among 456 infants without a Bayley-III assessment, 248 were ineligible due to language other than English , and the parent declined the assessment for 71. The Bayley-III analysis included 1006 infants whose mothers received an INSTI-based (n = 306), PI-based (n = 473), or NNRTI-based (n = 227) combination ART regimen. Overall, 531 infants (52.8%) were exposed to ART from conception onward. Demographic and maternal characteristics by maternal regimen are presented in Table 1; characteristics by ART timing are given in eTable 1 in Supplement 1.

**Table 1.** Demographic and Maternal Characteristics of Infants in the SMARTT Study Population for Bayley-III Analysis

Characteristic	Overall population, No. (%) (N = 1006)^a^	Initial maternal ART regimen in pregnancy, No. (%)^a^		
		INSTI-based (n = 306)	PI-based (n = 473)	NNRTI-based (n = 227)
**Maternal**				
Mother’s age at delivery, mean (SD), y	30.3 (6.0)	30.6 (5.8)	30.1 (6.1)	30.4 (6.1)
Race				
American Indian or Alaska Native	2 (0.2)	0	1 (0.2)	1 (0.4)
Asian	8 (0.8)	3 (1.0)	5 (1.1)	0
Black	786 (78.1)	233 (76.1)	364 (77.0)	189 (83.3)
Native Hawaiian or Other Pacific Islander	1 (0.1)	0	1 (0.2)	0
White	146 (14.5)	52 (17.0)	71 (15.0)	23 (10.1)
Multiracial	52 (5.2)	14 (4.6)	26 (5.5)	12 (5.3)
Not reported	11 (1.1)	4 (1.3)	5 (1.1)	2 (0.9)
Ethnicity				
Hispanic or Latino	168 (16.7)	54 (17.6)	83 (17.5)	31 (13.7)
Non-Hispanic or non-Latino	833 (82.8)	249 (81.4)	389 (82.2)	195 (85.9)
>1 Ethnicity	2 (0.2)	1 (0.3)	1 (0.2)	0
Not reported	3 (0.3)	2 (0.7)	0	1 (0.4)
Maternal educational level less than high school	248 (24.7)	67 (21.9)	114 (24.1)	67 (29.5)
Annual household income <$20 000	663 (65.9)	195 (63.7)	316 (66.8)	152 (67.0)
Employed	276 (27.4)	83 (27.1)	126 (26.6)	67 (29.5)
Housing situation				
Own house or apartment	132 (13.1)	47 (15.4)	58 (12.3)	27 (11.9)
Rent house or apartment	674 (67.0)	200 (65.4)	314 (66.4)	160 (70.5)
Other	200 (19.9)	59 (19.3)	101 (21.4)	40 (17.6)
Primary language				
English	773 (76.8)	237 (77.5)	364 (77.0)	172 (75.8)
Spanish	29 (2.9)	8 (2.6)	13 (2.7)	8 (3.5)
Bilingual	70 (7.0)	18 (5.9)	36 (7.6)	16 (7.0)
Other or not reported	134 (13.3)	43 (14.1)	60 (12.7)	31 (13.7)
Region				
Northeast	223 (22.2)	61 (19.9)	103 (21.8)	59 (26.0)
Midwest	134 (13.3)	50 (16.3)	72 (15.2)	12 (5.3)
South or Puerto Rico	481 (47.8)	141 (46.1)	229 (48.4)	111 (48.9)
West	168 (16.7)	54 (17.6)	69 (14.6)	45 (19.8)
Maternal substance use in first trimester				
Tobacco	158 (15.7)	46 (15.0)	92 (19.5)	20 (8.8)
Alcohol	66 (6.6)	18 (5.9)	40 (8.5)	8 (3.5)
Marijuana	103 (10.2)	36 (11.8)	49 (10.4)	18 (7.9)
Any illicit drug use	113 (11.2)	40 (13.1)	52 (11.0)	21 (9.3)
Perinatal acquisition of HIV	125 (12.4)	43 (14.1)	56 (11.8)	26 (11.5)
Maternal mental health diagnosis^b^	104 (10.3)	50 (16.3)	30 (6.3)	24 (10.6)
Earliest CD4 count in pregnancy, median (IQR)	526 (342-757)	517 (345-780)	507 (321-732)	599 (435-785)
Earliest CD4 <250 cells/mL	135 (13.4)	45 (14.7)	69 (14.6)	21 (9.3)
Earliest HIV VL <50 copies/mL	470 (46.7)	154 (50.3)	178 (37.6)	138 (60.8)
Duration taking initial regimen, median (IQR), wk	29.4 (19,3-37.7)	30.1 (19.4-37.9)	28.1 (19.6-37.4)	31.1 (18.9-38.1)
Continued same regimen during pregnancy	836 (83.1)	257 (84.0)	400 (84.6)	179 (78.9)
**Infant**				
Birth cohort				
2012-2015	521 (51.8)	66 (21.6)	332 (70.2)	123 (54.2)
2016-2019	350 (34.8)	144 (47.1)	126 (26.6)	80 (35.2)
2020-2023	135 (13.4)	96 (31.4)	15 (3.2)	24 (10.6)
Infant age at assessment, mean (SD), y	1.1 (0.1)	1.1 (0.1)	1.1 (0.1)	1.1 (0.1)
Sex				
Female	499 (49.6)	146 (47.7)	246 (52.0)	107 (47.1)
Male	507 (50.4)	160 (52.3)	227 (48.0)	120 (52.9)
Preterm birth (<37 weeks)	144 (14.3)	44 (14.4)	69 (14.6)	31 (13.7)
Received early intervention services	37 (3.7)	16 (5.2)	14 (3.0)	7 (3.1)

Abbreviations: ART, antiretroviral therapy; Bayley-III, Bayley Scales of Infant and Toddler Development–3rd Edition; INSTI, integrase strand transfer inhibitor; NNRTI, nonnucleoside reverse transcriptase inhibitor; PI, protease inhibitor; SMARTT, Surveillance Monitoring for ART Toxicities; VL, viral load.

^a^ Note that some participants had missing data on certain characteristics, and percentages are reported among all those in category. The number with measurements not available or not reported was race (n = 11), ethnicity (n = 3), educational level (n = 22), household income (n = 69), employment status (n = 12), maternal substance use (n = 23 to 27), perinatal HIV status (n = 7), CD4 count (n = 26), HIV viral load (n = 11), preterm birth (n = 5) and receipt of early intervention services (n = 3).

^b^ Includes depression, anxiety, bipolar disorder, schizophrenia, posttraumatic stress disorder, and attention-deficit/hyperactivity disorder, reported before or during pregnancy.

Among the mothers of 1006 children, the mean (SD) maternal age at delivery was 30.3 (6.0) years, 663 women (65.9%) had an annual household income of less than $20 000, 248 (24.7%) had not completed high school, and 125 (12.4%) had perinatally acquired HIV. In terms of race, 2 mothers (0.2%) were American Indian or Alaska Native; 8 (0.8%), Asian; 786 (78.1%), Black or African American; 1 (0.1%), Native Hawaiian or other Pacific Islander; 146 (14.5%), White; 52 (5.2%), multiracial; and 11 (1.1%) did not report their race. For ethnicity, 168 mothers (16.7%) were Hispanic or Latino, 833 (82.8%) were non-Hispanic, 2 (0.2%) reported more than 1 ethnicity, and 3 did not report ethnicity. Based on earliest pregnancy measures, the median CD4 count was 526 cells/mL but only 470 women (46.7%) had suppressed VL (<50 copies/mL). The prevalence of self-reported substance use during the first trimester included 158 women (15.7%) reporting tobacco use, 103 (10.2%) reporting marijuana use, and 66 (6.6%) reporting alcohol use. Most mothers (836 [83.1%) continued the same regimen throughout pregnancy (median duration, 29.4 [IQR, 19.3-37.7] weeks).

While most characteristics were similar by regimen, a higher percentage of mothers initiating NNRTI-based regimens were Black (199 of 227 [87.7%]) and had lower educational levels (67 of 227 [29.5%] did not complete high school), while a lower proportion lived in the Midwest (12 of 227 [5.3%]). Mothers initiating NNRTI-based regimens also less often reported first trimester tobacco (20 of 227 [8.8%]) or alcohol (8 of 227 [3.5%]) use. Maternal VL was more often suppressed for mothers initiating NNRTI-based regimens (138 of 227 [60.8%]) than PI- or INSTI-based regimens (178 of 473 [37.6%] and 154 of 306 [50.3%], respectively). Characteristics were generally comparable by timing of ART initiation (eTable 1 in Supplement 1). Infants included in the analysis were less often Hispanic (168 of 1006 [16.7%]) than those without a Bayley-III assessment (159 of 641 [24.8%]), but had similar maternal age, income and educational levels, and rates of substance use and preterm birth as those who were enrolled but had no 1-year visit (eTable 2 in Supplement 1).

Given its availability in both Spanish and English, more infants had an MCDI assessment; of 1363 infants assessed, 1160 had maternal exposure to an INSTI-based (n = 347), PI-based (n = 546), or NNRTI-based (n = 267) combination regimen (eFigure 1 in Supplement 1). The distribution of demographic and maternal characteristics was similar to that of infants with the Bayley-III assessments, although the proportion of Hispanic infants was higher (341 of 1160 [29.4%]) (eTable 3 in Supplement 1).

### Comparison of Bayley-III Scores by Maternal ART Regimen

**Figure 2.**
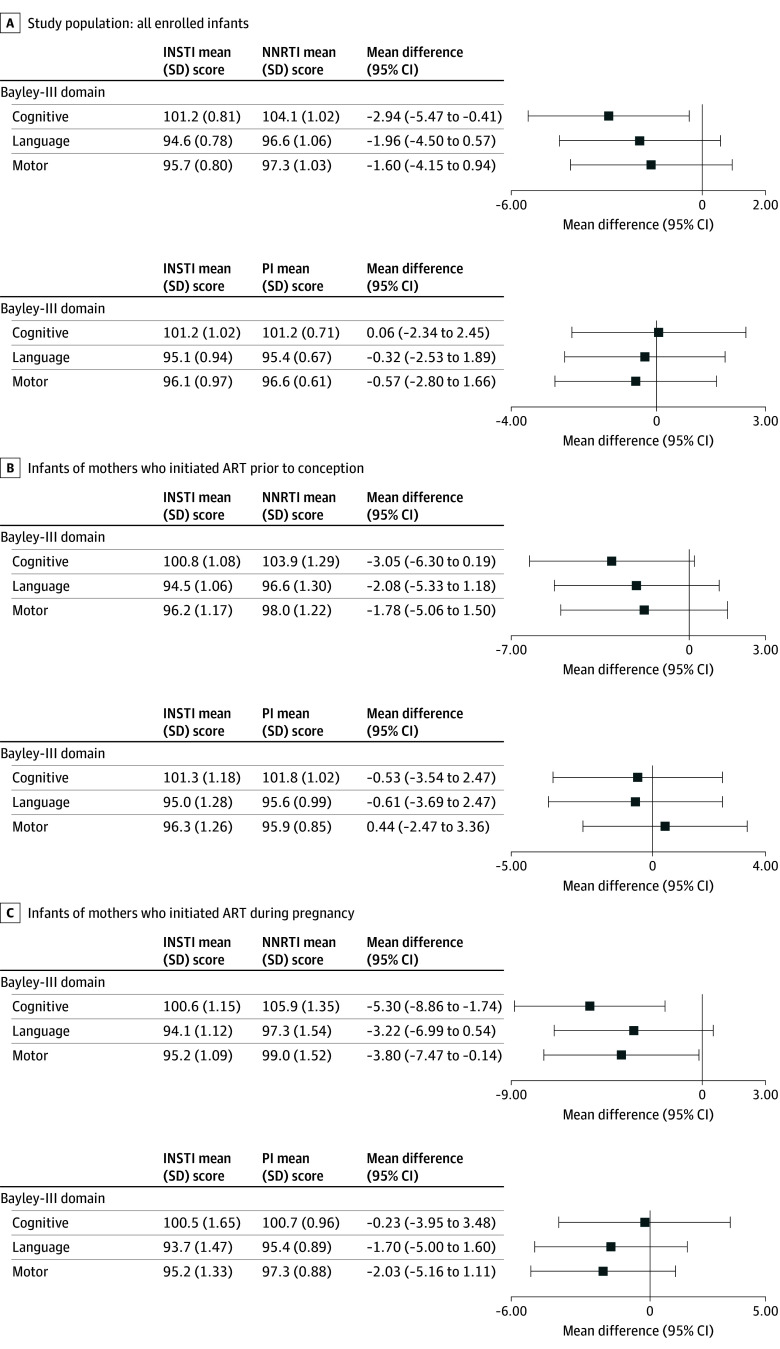
Adjusted Mean Differences in Bayley Scales of Infant and Toddler Development–3rd Edition (Bayley-III) Scores by In Utero Exposure to Initial Maternal Antiretroviral Therapy (ART) Regimen Doubly robust estimates of mean differences in Bayley-III scores between infants with in utero exposure to integrase strand transfer inhibitor (INSTI)–based compared with nonnucleoside reverse transcriptase inhibitor (NNRTI)–based or protease inhibitor (PI)–based regimens. Analyses accounted for the following covariates in regression adjustment and in inverse probability weighting for treatment regimen: maternal educational attainment, household income level, birth year (categorized as 2012-2015, 2016-2019, or 2020-2023), maternal perinatal HIV status, geographic region, maternal mental health diagnosis, maternal age at delivery, maternal substance use in the first trimester (alcohol, tobacco, or marijuana, each considered separately), and for regression adjustment only, sex at birth.

Overall, mean (SD) scores for cognitive (101.7 [14.2]), language (95.5 [13.8]), and motor development (96.4 [13.2]) domains were near reference standards (100 [15]). Unadjusted mean scores by initial maternal regimen are presented in eTable 4 in Supplement 1. Doubly robust estimates of differences in mean Bayley-III scores (Figure 2 and Table 2) indicated no significant difference in cognitive, language, or motor development domain scores between infants exposed to INSTI-based vs PI-based regimens. However, mean cognitive scores for INSTI-exposed infants were 2 to 3 points lower than those for NNRTI-exposed infants (adjusted mean difference, −2.94; 95% CI, −5.47 to −0.41; *P* = .02). These differences were more pronounced among infants whose mothers initiated ART during pregnancy. Notably, adjusted mean cognitive scores for INSTI-exposed infants were close to the expected population mean of 100, while NNRTI-exposed infants scored 4 to 5 points above this reference mean (adjusted mean difference, −5.30; 95% CI, −8.86 to −1.74; *P* = .004).

**Table 2.** Doubly Robust Estimates of Mean Differences in Bayley-III Scores by In Utero Exposure to Initial Maternal ART Regimen

Bayley-III domain by comparison	Initial maternal ART regimen in pregnancy, estimated mean (SE)			Adjusted mean difference (95% CI)^a^	*P* value
	INSTI-based	PI-based	NNRTI-based		
**All enrolled infants**					
No. of infants	301	466	227	NA	NA
INSTI vs NNRTI					
Cognitive	101.19 (0.81)	NA	104.13 (1.02)	−2.94 (−5.47 to −0.41)	.02
Language	94.63 (0.78)	NA	96.60 (1.06)	−1.96 (−4.50 to 0.57)	.13
Motor development	95.71 (0.80)	NA	97.32 (1.03)	−1.60 (−4.15 to 0.94)	.22
INSTI vs PI					
Cognitive	101.23 (1.02)	101.17 (0.71)	NA	0.06 (−2.34 to 2.45)	.96
Language	95.08 (0.94)	95.40 (0.67)	NA	−0.32 (−2.53 to 1.89)	.77
Motor development	96.07 (0.97)	96.65 (0.61)	NA	−0.57 (−2.80 to 1.66)	.61
**Infants born to mothers using ART at conception**					
No. of infants	167	216	142	NA	NA
INSTIs vs NNRTIs					
Cognitive	100.80 (1.08)	NA	103.86 (1.29)	−3.05 (−6.30 to 0.19)	.07
Language	94.51 (1.06)	NA	96.58 (1.30)	−2.08 (−5.33 to 1.18)	.21
Motor development	96.20 (1.17)	NA	97.99 (1.22)	−1.78 (−5.06 to 1.50)	.29
INSTIs vs PIs					
Cognitive	101.29 (1.18)	101.82 (1.02)	NA	−0.53 (−3.54 to 2.47)	.73
Language	95.03 (1.28)	95.64 (0.99)	NA	−0.61 (−3.69 to 2.47)	.70
Motor development	96.31 (1.26)	95.87 (0.85)	NA	0.44 (−2.47 to 3.36)	.77
**Infants born to mothers who initiated ART during pregnancy**					
No. of infants	134	250	85	NA	NA
INSTIs vs NNRTIs					
Cognitive	100.61 (1.15)	NA	105.91 (1.35)	−5.30 (−8.86 to −1.74)	.004
Language	94.12 (1.12)	NA	97.34 (1.54)	−3.22 (−6.99 to 0.54)	.09
Motor development	95.21 (1.09)	NA	99.01 (1.52)	−3.80 (−7.47 to −0.14)	.04
INSTIs vs PIs					
Cognitive	100.46 (1.65)	100.69 (0.96)	NA	−0.23 (−3.95 to 3.48)	.90
Language	93.67 (1.47)	95.37 (0.89)	NA	−1.70 (−5.00 to 1.60)	.31
Motor development	95.23 (1.33)	97.25 (0.88)	NA	−2.03 (−5.16 to 1.11)	.20

Abbreviations: ART, antiretroviral therapy; Bayley-III, Scales of Infant and Toddler Development–3rd Edition; INSTI, integrase strand transfer inhibitor; NNRTI, nonnucleoside reverse transcriptase inhibitor; NA, not applicable; PI, protease inhibitor.

^a^ Mean estimated causal effect from doubly robust estimators, accounting for the following covariates in regression adjustment and in inverse probability weighting for treatment regimen: maternal educational level, household income level, birth year (categorized as 2012-2015, 2016-2019, or 2020-2023), maternal perinatal HIV status, maternal mental health diagnosis, geographic region, maternal age at delivery, maternal substance use in the first trimester (alcohol, tobacco, or marijuana, each considered separately), and for regression adjustment only, sex at birth.

The 1006 infants with Bayley-III assessments were born to 849 unique mothers in 987 pregnancies (including 38 twins, 290 siblings). Sensitivity analyses accounting for correlation in outcomes among siblings yielded similar results to those of the primary analyses, with no difference between INSTI- vs PI-based regimens but a 3- to 4-point lower mean cognitive score for INSTI- vs NNRTI-based regimens (adjusted mean difference, −2.50; 95% CI, −4.98 to −0.01; *P* = 0.49) (eTable 5 in Supplement 1). Analyses restricted to infants whose mothers continued the same regimen throughout pregnancy yielded similar findings (eTable 6 in Supplement 1). Adjusting for maternal CD4 level and suppressed VL resulted in attenuated differences between INSTI- and NNRTI-based regimens (eTable 7 in Supplement 1). When evaluating differences in outcomes by trimester of ART initiation, no meaningful difference in scores was observed for NNRTI- or PI-based regimens. However, infants whose mothers initiated INSTI-based regimens in the first trimester had mean scores that were about 3 points lower across all 3 Bayley-III domains than INSTI-unexposed infants, while those exposed later in pregnancy (second or third trimester) had slightly higher mean scores (eTable 8 in Supplement 1).

In exploratory analyses evaluating exposure to individual INSTI medications, accounting for all antiretroviral medications in maternal regimens during pregnancy, we observed no association of any individual INSTI (elvitegravir, dolutegravir, raltegravir, or bictegravir) or INSTI exposure overall with Bayley-III cognitive or language scores. Slightly lower motor development scores were observed across individual INSTIs, with estimated mean differences for exposed vs unexposed infants close to the INSTI drug class mean (−1.84; 95% CI, −3.83 to 0.14; *P* = .07), although exposure rates were low (4%-15%) (eTables 9-11 in Supplement 1). Fixed-effect models including all individual antiretroviral medications simultaneously yielded similar conclusions, although the estimates varied more within each drug class and had wider 95% CIs.

### Comparison of Low MCDI Indicators by Maternal ART Regimen

Among 1160 infants with an MCDI assessment, the proportion scoring at or less than the 10th percentile included 188 of 1128 (16.7%) for A-E total gestures, 85 of 1128 (7.5%) for phrases understood, 158 of 1128 (14.0%) for vocabulary comprehension, and 76 of 1128 (6.7%) for word production. MCDI summary statistics by initial maternal ART regimen are provided in eTable 4 in Supplement 1. There was no difference in the adjusted prevalence of infants with low MCDI scores between those exposed to INSTI-based regimens compared with either PI- or NNRTI-based regimens for 3 domains (Table 3). However, for the phrases understood domain, INSTI-exposed infants had a 4.46% (95% CI, 0.06%-8.86%) higher adjusted prevalence of low scores than NNRTI-exposed infants and a 5.08% (95% CI, −0.21% to 10.38%) higher adjusted prevalence of low scores than PI-exposed infants. These differences were similar among infants whose mothers initiated ART before conception, but no difference between INSTI-exposed and NNRTI-exposed infants was observed among those whose mothers initiated ART during pregnancy (Table 3).

**Table 3.** Doubly Robust Estimates of Risk Differences in Indicators of MCDI at or Below the 10th Percentile by In Utero Exposure to Initial Maternal ART Regimen

MCDI domain by comparison MCDI domain	Initial maternal ART regimen in pregnancy, estimated mean (SE)			Adjusted risk difference (95% CI)^a^	*P* value
	INSTI-based	PI-based	NNRTI-based		
**All enrolled infants**					
No. of infants	339	527	62	NA	NA
INSTIs vs NNRTIs					
A-E total gestures	17.45 (2.15)	NA	16.43 (2.37)	1.02 (−5.18 to 7.22)	.75
Phrases understood	10.21 (1.79)	NA	5.75 (1.45)	4.46 (0.06 to 8.86)	.047
Vocabulary comprehension	13.98 (2.00)	NA	13.46 (2.05)	0.52 (−5.01 to 6.05)	.85
Word production	6.01 (1.45)	NA	6.41 (1.49)	−0.40 (−4.41 to 3.61)	.85
INSTIs vs PIs					
A-E total gestures	17.48 (2.45)	14.91 (1.73)	NA	2.58 (−3.26 to 8.42)	.39
Phrases understood	12.04 (2.36)	6.96 (1.35)	NA	5.08 (−0.21 to 10.38)	.06
Vocabulary comprehension	14.16 (2.34)	13.20 (1.81)	NA	0.97 (−4.78 to 6.71)	.74
Word production	4.99 (1.58)	8.77 (1.68)	NA	−3.78 (−8.27 to 0.71)	.10
**Infants of mothers who initiated ART prior to conception**					
No. of infants	182	242	165	NA	NA
INSTIs vs NNRTIs					
A-E total gestures	19.83 (3.10)	NA	17.57 (2.89)	2.25 (−5.91 to 10.42)	.59
Phrases understood	11.26 (2.61)	NA	6.27 (1.87)	4.99 (−1.08 to 11.05)	.11
Vocabulary comprehension	16.28 (2.99)	NA	13.75 (2.51)	2.53 (−4.99 to 10.05)	.51
Word production	6.13 (1.67)	NA	7.63 (1.86)	−1.50 (−6.20 to 3.20)	.53
INSTIs vs PIs					
A-E total gestures	21.47 (3.52)	18.22 (3.46)	NA	3.25 (−6.25 to 12.74)	.50
Phrases understood	13.31 (3.12)	7.57 (1.63)	NA	5.74 (−1.05 to 12.54)	.10
Vocabulary comprehension	16.61 (3.19)	16.15 (3.68)	NA	0.46 (−8.98 to 9.90)	.92
Word production	4.58 (1.52)	7.05 (1.66)	NA	−2.46 (−6.91 to 1.98)	.28
**Infants of mothers who initiated ART during pregnancy**					
No. of infants	157	285	97	NA	NA
INSTIs vs NNRTIs					
A-E total gestures	13.69 (2.82)	NA	12.46 (5.18)	1.22 (−10.31 to 12.76)	.84
Phrases understood	9.80 (2.39)	NA	10.62 (1.93)	−0.82 (−6.83 to 5.19)	.79
Vocabulary comprehension	14.49 (3.52)	NA	16.58 (3.20)	−2.09 (−11.33 to 7.15)	.66
Word production	9.80 (2.39)	NA	10.62 (1.93)	−0.82 (−6.83 to 5.19)	.79
INSTIs vs PIs					
A-E total gestures	13.45 (3.66)	12.77 (1.99)	NA	0.67 (−7.54 to 8.89)	.87
Phrases understood	11.72 (3.46)	5.21 (1.61)	NA	6.51 (−0.93 to 13.94)	.09
Vocabulary comprehension	12.85 (3.78)	11.53 (2.14)	NA	1.32 (−7.16 to 9.80)	.76
Word production	8.01 (3.23)	7.90 (2.32)	NA	0.11 (−7.69 to 7.90)	.98

Abbreviations: ART, antiretroviral therapy; INSTI, integrase strand inhibitor; NNRTI, nonnucleoside reverse transcriptase inhibitor; NA, not applicable; PI, protease inhibitor.

^a^ Mean estimated causal effect from doubly robust estimators, accounting for the following covariates in regression adjustment and in inverse probability weighting for treatment regimen: maternal educational level, household income level, birth year (categorized as 2012-2015, 2016-2019, or 2020-2023), maternal perinatal HIV status, maternal mental health diagnosis, geographic region, maternal age at delivery, maternal substance use in the first trimester (alcohol, tobacco, or marijuana, each considered separately), and for regression adjustment only, sex at birth.

## Discussion

In this large US-based cohort of uninfected infants born to mothers with HIV, we found little evidence of clinically meaningful neurodevelopmental differences at 1 year of age among those with in utero exposure to INSTI-based regimens vs those exposed to PI- or NNRTI-based regimens. Mean Bayley-III scores for each domain were close to the standard population mean of 100 within each regimen exposure group, and fewer than 8% had MCDI scores at or below the 10th population percentile for the phrases understood and word production domains, indicating lower than expected levels of impairment. While the percentage with low MCDI scores in the other 2 domains was above 10% (14.0% and 16.7%), the adjusted prevalence was similar by maternal ARV exposure groups. These findings highlight the overall resilience and neurodevelopmental health of these children, despite often living in families faced with socioeconomic and other social challenges. As one of the first studies to examine neurodevelopment with respect to in utero exposure to INSTI-based regimens, the results support the continued recommendation of INSTIs as part of first-line treatment for pregnant women with HIV.^13,14^

We observed no indication of a difference in mean Bayley-III cognitive, language, or motor development scores between those exposed to INSTI-based vs PI-based regimens, and only very small mean differences (approximately 3 points) for those exposed to INSTI-based regimens compared with NNRTI-based regimens. These differences were more pronounced among infants born to mothers who initiated ART during pregnancy than for those born to mothers who were taking ART at conception. The largest mean differences of about 5 points were for cognitive scores, but the adjusted mean scores for INSTI-exposed infants were very close to the expected population mean of 100, while those for NNRTI-exposed infants were 4 to 5 points above this reference standard.

Sensitivity analyses provided consistent estimates and indicated that maternal health factors, particularly the higher rate of VL suppression among mothers initiating NNRTI-based regimens and those taking ART at conception, may partially explain the differences between INSTI-exposed and NNRTI-exposed infants and by timing of ART initiation. There was no evidence that later initiation of INSTI-based ART adversely impacted development; infants whose mothers started taking INSTIs in the second or third trimester did not have lower mean scores than those exposed to INSTIs from conception.

MCDI-based assessments also showed few adverse differences for INSTI-exposed infants compared with other ART regimens. Similar to the findings for Bayley-III assessments, the only difference observed—higher prevalence of low phrases understood scores in INSTI-exposed infants—was driven by lower-than-expected prevalences of low scores among NNRTI-exposed infants rather than deficits in the INSTI group. These results suggest that language development in our cohort remained largely unaffected by type of maternal ART.

### Strengths and Limitations

Our analysis has several strengths that bolster the validity of our findings. The SMARTT study is one of the largest prospective cohorts of infants exposed to but not infected with HIV to be followed up throughout childhood, with detailed neurodevelopmental assessments conducted by trained neuropsychologists. The study collects a rich set of covariates related to maternal health and pregnancy exposures, demographic characteristics, and home environment, for which adjusting can reduce possible confounding. The doubly robust statistical approach we used accounted for differences in the characteristics of mothers initiating different types of regimens, as well as individual characteristics that might predict neurodevelopment.^23,24,29^ The hierarchical mixed-effect model used to evaluate individual antiretroviral medications has been demonstrated to yield less biased and more precise estimates than fitting a model with fixed effects for every individual antiretroviral medication.^5,25,30,31^ Nonetheless, given the limited numbers exposed to specific INSTI medications, these findings warrant further confirmation.

However, we recognize some limitations in our study. Our analysis did not include a comparison cohort of infants who were not exposed to HIV, so we were unable to directly compare neurodevelopment of infants who were and were not exposed.^11,12^ Although we adjusted for a wide range of covariates anticipated to be related to both maternal ART regimens and neurodevelopment, the possibility of residual confounding from unmeasured factors remains. We expected misclassification of ART exposure to be rare and unrelated to neurodevelopmental outcomes; however, such bias could attenuate estimated treatment differences. The greater diversity of ART regimens prescribed in the US compared with other regions may complicate accurately predicting the probabilities of receiving one regimen vs another, used in calculating weights for doubly robust estimators.^32^ The lack of access to a Spanish-language Bayley-III assessment also makes our findings less generalizable to Hispanic populations.

## Conclusions

In this prospective cohort study, we observed little evidence of clinically meaningful differences in neurodevelopment for infants exposed to INSTI-based regimens during gestation compared with combination regimens that are PI-based or NNRTI-based. Our findings support current recommendations to include INSTI-based regimens as part of first-line treatment for pregnant women with HIV, whether women are already taking ART at conception or initiating ART during pregnancy. Ongoing surveillance of long-term outcomes in infants who are exposed to HIV but are not infected remains essential as treatment guidelines evolve, and further evaluation of individual INSTI medications should be prioritized as the numbers exposed continue to increase.

## Notes

Supplement 1. **eTable 1.** Demographic and Maternal Characteristics of Infants in SMARTT Study Population for Bayley-III Analysis, by Timing of Initial Maternal ARV Regimen During Pregnancy **eTable 2.** Maternal and Background Characteristics by Inclusion Status **eTable 3.** Demographic and Maternal Characteristics of Infants in SMARTT Study Population for MCDI Analysis, by Initial Maternal ARV Regimen During Pregnancy **eTable 4.** Summary Statistics for Bayley-III and MCDI Scores, by Initial Maternal ARV Regimen **eTable 5.** Sensitivity Analysis Results for Doubly Robust Estimates of Mean Differences in Bayley-III Scores by In Utero Exposure to Initial Maternal ART Regimen, Accounting for Correlated Outcomes Among Children Born to the Same Mother **eTable 6.** Sensitivity Analysis Results for Doubly Robust Estimates of Mean Differences in Bayley-III Scores by In Utero Exposure to Initial Maternal ART Regimen, Restricted to Infants of Mothers Who Continued the Same Regimen Type Throughout Pregnancy **eTable 7.** Sensitivity Analysis Results for Doubly Robust Estimates of Mean Differences in Bayley-III Scores by In Utero Exposure to Initial Maternal ART Regimen, Further Adjusted for Maternal CD4 and VL During Pregnancy **eTable 8.** Effect of Timing of ARV Initiation Within Drug Class on Bayley-III Outcomes, Unadjusted and Adjusted for Covariates **eTable 9.** Association of In Utero Exposure to Individual ARV Medications at Any Time During Gestation With Bayley-III Cognitive Scores Using a Hierarchical Model and a Full Model **eTable 10.** Association of In Utero Exposure to Individual ARV Medications at Any Time During Gestation With Bayley-III Language Scores Using a Hierarchical Model and a Full Model **eTable 11.** Association of In Utero Exposure to Individual ARV Medications at Any Time During Gestation With Bayley-III Motor Development Scores Using a Hierarchical Model and a Full Model **eFigure.** Derivation of Study Population for MCDI Neurodevelopmental Analysis **eAppendix.** Additional Acknowledgments

Supplement 2.
Data Sharing Statement
