# Supplementary material for: Maternal Use of Integrase Strand Transfer Inhibitors During Pregnancy and Infant Neurodevelopment
Source: JAMA Netw Open. 2025 Nov 26;8(11):e2545652. doi: 10.1001/jamanetworkopen.2025.45652 (PMC12658668; doi:10.1001/jamanetworkopen.2025.45652)
Supplement: Supplement 1. — eTable 1. Demographic and Maternal Characteristics of Infants in SMARTT Study Population for Bayley-III Analysis, by Timing of Initial Maternal ARV Regimen During Pregnancy eTable 2. Maternal and Background Characteristics by Inclusion Status eTable 3. Demographic and Maternal Characteristics of Infants in SMARTT Study Population for MCDI Analysis, by Initial Maternal ARV Regimen During Pregnancy eTable 4. Summary Statistics for Bayley-III and MCDI Scores, by Initial Maternal ARV Regimen eTable 5. Sensitivity Analysis Results for Doubly Robust Estimates of Mean Differences in Bayley-III Scores by In Utero Exposure to Initial Maternal ART Regimen, Accounting for Correlated Outcomes Among Children Born to the Same Mother eTable 6. Sensitivity Analysis Results for Doubly Robust Estimates of Mean Differences in Bayley-III Scores by In Utero Exposure to Initial Maternal ART Regimen, Restricted to Infants of Mothers Who Continued the Same Regimen Type Throughout Pregnancy eTable 7. Sensitivity Analysis Results for Doubly Robust Estimates of Mean Differences in Bayley-III Scores by In Utero Exposure to Initial Maternal ART Regimen, Further Adjusted for Maternal CD4 and VL During Pregnancy eTable 8. Effect of Timing of ARV Initiation Within Drug Class on Bayley-III Outcomes, Unadjusted and Adjusted for Covariates eTable 9. Association of In Utero Exposure to Individual ARV Medications at Any Time During Gestation With Bayley-III Cognitive Scores Using a Hierarchical Model and a Full Model eTable 10. Association of In Utero Exposure to Individual ARV Medications at Any Time During Gestation With Bayley-III Language Scores Using a Hierarchical Model and a Full Model eTable 11. Association of In Utero Exposure to Individual ARV Medications at Any Time During Gestation With Bayley-III Motor Development Scores Using a Hierarchical Model and a Full Model eFigure. Derivation of Study Population for MCDI Neurodevelopmental Analysis eAppendix. Additional Acknowledgments [file jamanetwopen-e2545652-s001.pdf]

Supplementary Online Content

Williams PL. Boahene M, Mash LE, et al. Maternal use of integrase strand transfer inhibitors during pregnancy and infant neurodevelopment. *JAMA Netw Open.* 2025;8(11):e2545652. doi:10.1001/jamanetworkopen.2025.45652

eTable 1. Demographic and Maternal Characteristics of Infants in SMARTT Study Population for Bayley-III Analysis, by Timing of Initial Maternal ARV Regimen During Pregnancy
eTable 2. Maternal and Background Characteristics by Inclusion Status
eTable 3. Demographic and Maternal Characteristics of Infants in SMARTT Study Population for MCDI Analysis, by Initial Maternal ARV Regimen During Pregnancy
eTable 4. Summary Statistics for Bayley-III and MCDI Scores, by Initial Maternal ARV Regimen
eTable 5. Sensitivity Analysis Results for Doubly Robust Estimates of Mean Differences in Bayley-III Scores by In Utero Exposure to Initial Maternal ART Regimen, Accounting for Correlated Outcomes Among Children Born to the Same Mother
eTable 6. Sensitivity Analysis Results for Doubly Robust Estimates of Mean Differences in Bayley-III Scores by In Utero Exposure to Initial Maternal ART Regimen, Restricted to Infants of Mothers Who Continued the Same Regimen Type Throughout Pregnancy
eTable 7. Sensitivity Analysis Results for Doubly Robust Estimates of Mean Differences in Bayley-III Scores by In Utero Exposure to Initial Maternal ART Regimen, Further Adjusted for Maternal CD4 and VL During Pregnancy
eTable 8. Effect of Timing of ARV Initiation Within Drug Class on Bayley-III Outcomes, Unadjusted and Adjusted for Covariates
eTable 9. Association of In Utero Exposure to Individual ARV Medications at Any Time During Gestation With Bayley-III Cognitive Scores Using a Hierarchical Model and a Full Model
eTable 10. Association of In Utero Exposure to Individual ARV Medications at Any Time During Gestation With Bayley-III Language Scores Using a Hierarchical Model and a Full Model
eTable 11. Association of In Utero Exposure to Individual ARV Medications at Any Time During Gestation With Bayley-III Motor Development Scores Using a Hierarchical Model and a Full Model
eFigure. Derivation of Study Population for MCDI Neurodevelopmental Analysis
eAppendix. Additional Acknowledgments

This supplementary material has been provided by the authors to give readers additional information about their work.

© 2025 Williams PL et al. *JAMA Network Open*.

eTable 1. Demographic and Maternal Characteristics of Infants in SMARTT Study Population for Bayley-III Analysis, by Timing of Initial Maternal ARV Regimen During Pregnancy

Characteristic		Total (N=1006)	Timing of Initial Maternal ARV Regimen in Pregnancy: During pregnancy (N=475)	Timing of Initial Maternal ARV Regimen in Pregnancy: At conception (N=531)
Maternal characteristics				
Mother's age at delivery, mean (SD)		30.3 (6.0)	29.6 (5.8)	31.0 (6.1)
Black race		828 (82%)	405 (85%)	423 (80%)
Hispanic ethnicity		168 (17%)	54 (18%)	83 (18%)
Low maternal education (< high school graduate)		248 (25%)	109 (23%)	139 (26%)
Annual household income < $20K		663 (66%)	335 (71%)	328 (62%)
Employed		276 (27%)	122 (26%)	154 (29%)
Living Situation				
	Owned house/apartment	132 (13%)	52 (11%)	80 (15%)
	Rented house/apartment	674 (67%)	318 (67%)	356 (67%)
	Other	200 (20%)	105 (22%)	95 (18%)
Primary Language				
	English	773 (77%)	388 (82%)	385 (73%)
	Spanish	29 (3%)	12 (3%)	17 (3%)
	Bilingual	70 (7%)	29 (6%)	41 (8%)
	Other/not reported	134 (13%)	46 (9%)	88 (16%)
Region	Northeast	223 (22%)	79 (17%)	144 (27%)
	Midwest	134 (13%)	66 (14%)	68 (13%)
	South or Puerto Rico	481 (48%)	262 (55%)	219 (41%)
	West	168 (17%)	68 (14%)	100 (19%)
Maternal substance use in First Trimester				
	Tobacco	158 (16%)	96 (20%)	62 (12%)
	Alcohol	66 (7%)	37 (8%)	29 (5%)
	Marijuana	103 (10%)	59 (12%)	44 (8%)
	Any Illicit Drug Use	113 (11%)	64 (13%)	49 (9%)
Perinatal acquisition of HIV		125 (12%)	51 (11%)	74 (14%)
Earliest CD4 count in pregnancy (median, IQR)		526 (342, 757)	458 (305, 658)	599 (417, 830)
Earliest CD4<250 cells/mL		135 (13.8%)	78 (16.8%)	57 (11.0%)
Earliest VL<50 copies/mL		470 (47.2%)	121 (25.7%)	349 (66.6%)
Infant characteristics				
Birth cohort				
	2012-2015	521 (52%)	269 (57%)	252 (47%)
	2016-2019	350 (35%)	160 (34%)	190 (36%)
	2020-2023	135 (13%)	46 (10%)	89 (17%)
Infant age at assessment (mean, SD)		1.1 (0.1)	1.1 (0.1)	1.1 (0.1)
Female sex		499 (50%)	230 (48%)	269 (51%)
Preterm birth (< 37 weeks)		144 (14%)	65 (14%)	79 (15%)
Received Early Intervention Services (EIS)		37 (4%)	19 (4%)	18 (3%)

© 2025 Williams PL et al. *JAMA Network Open*.

eTable 2. Maternal and Background Characteristics by Inclusion Status

Characteristic	Total (N=2271)	Inclusion Status in Analysis: Included in analysis (N=1006)	Had age 1 visit, but no ND assessment or not on regimen of interest (N=624)	Enrolled, but no age 1 visit (N=641)
Mothers age at delivery, mean (SD)	30.1 (6.0)	30.3 (6.0)	30.1 (6.1)	29.8 (5.9)
Black race	1,611 (70.9%)	828 (82.3%)	318 (51.0%)	465 (72.5%)
Hispanic ethnicity	627 (27.6%)	168 (16.7%)	300 (48.1%)	159 (24.8%)
Low maternal education (< high school)	582 (25.6%)	248 (24.7%)	172 (27.6%)	162 (25.3%)
Annual household income < $20K	1,454 (64.0%)	664 (66.0%)	428 (68.6%)	362 (56.5%)
Employed	596 (26.2%)	278 (27.6%)	140 (22.4%)	178 (27.8%)
Primary Language				
English	1,115 (49.1%)	772 (76.7%)	263 (42.1%)	80 (12.5%)
Spanish	233 (10.3%)	29 (2.9%)	186 (29.8%)	18 (2.8%)
Bilingual	138 (6.1%)	70 (7.0%)	55 (8.8%)	13 (2.0%)
Other	206 (9.1%)	101 (10.0%)	97 (15.5%)	8 (1.2%)
Not reported	579 (25.5%)	34 (3.4%)	23 (3.7%)	522 (81.4%)
Region				
Northeast	429 (18.9%)	223 (22.2%)	97 (15.5%)	109 (17.0%)
Midwest	217 (9.6%)	134 (13.3%)	31 (5.0%)	52 (8.1%)
South	1,044 (46.0%)	479 (47.6%)	214 (34.3%)	351 (54.8%)
West	444 (19.6%)	168 (16.7%)	172 (27.6%)	104 (16.2%)
Puerto Rico	137 (6.0%)	2 (0.2%)	110 (17.6%)	25 (3.9%)
Perinatal HIV	326 (14.4%)	125 (12.4%)	122 (19.6%)	79 (12.3%)
Maternal substance use in first trimester				
Tobacco	321 (14.1%)	158 (15.7%)	65 (10.4%)	98 (15.3%)
Alcohol	160 (7.0%)	66 (6.6%)	47 (7.5%)	47 (7.3%)
Marijuana	202 (8.9%)	103 (10.2%)	38 (6.1%)	61 (9.5%)
Any Illicit Drug Use	231 (10.2%)	113 (11.2%)	44 (7.1%)	74 (11.5%)
Infant Birth cohort				
2012-2015	1,006 (44.3%)	521 (51.8%)	271 (43.4%)	214 (33.4%)
2016-2019	819 (36.1%)	350 (34.8%)	222 (35.6%)	247 (38.5%)
2020-2023	446 (19.6%)	135 (13.4%)	131 (21.0%)	180 (28.1%)
Female sex	1,085 (47.8%)	499 (49.6%)	278 (44.6%)	308 (48.0%)
Preterm birth (< 37 weeks)	352 (15.5%)	144 (14.3%)	100 (16.0%)	108 (16.8%)

© 2025 Williams PL et al. *JAMA Network Open*.

eTable 3. Demographic and Maternal Characteristics of Infants in SMARTT Study Population for MCDI Analysis, by Initial Maternal ARV Regimen During Pregnancy

Characteristic	Total (N=1160)	Initial Maternal ARV Regimen in Pregnancy<br>INSTI-based regimen (N=347)	PI-based regimen (N=546)	NNRTI-based regimen (N=267)
Maternal characteristics				
Mother's age at delivery, mean (SD)	30.3 (5.9)	30.7 (5.8)	30.0 (6.0)	30.3 (6.0)
Black race	820 (71%)	237 (68%)	388 (71%)	195 (73%)
Hispanic ethnicity	341 (29%)	105 (30%)	161 (29%)	75 (28%)
Low maternal education (< high school graduate)	296 (26%)	76 (22%)	141 (26%)	79 (30%)
Annual household income < $20K	792 (68%)	226 (65%)	380 (70%)	186 (70%)
Employed	303 (26%)	95 (27%)	136 (25%)	72 (27%)
Living Situation				
Own house/apartment	157 (14%)	46 (13%)	79 (14%)	32 (12%)
Rent house/apartment	763 (66%)	225 (65%)	352 (64%)	186 (70%)
Other	240 (21%)	76 (22%)	115 (21%)	49 (18%)
Primary Language				
English	783 (68%)	236 (68%)	371 (68%)	176 (66%)
Spanish	156 (13%)	50 (14%)	68 (12%)	38 (14%)
Bilingual	85 (7%)	22 (6%)	42 (8%)	21 (8%)
Other/not reported	136 (12%)	39 (11%)	65 (12%)	32 (12%)
Region Northeast	213 (18%)	53 (15%)	101 (18%)	59 (22%)
Midwest	126 (11%)	46 (13%)	70 (13%)	10 (4%)
South or Puerto Rico	581 (50%)	170 (48%)	283 (52%)	128 (48%)
West	240 (21%)	78 (22%)	92 (17%)	70 (26%)
Perinatal acquisition of HIV	146 (13%)	47 (14%)	68 (12%)	31 (12%)
Maternal mental health diagnosis	111 (10%)	54 (16%)	31 (6%)	26 (10%)
Maternal substance use in First Trimester				
Tobacco	170 (15%)	45 (13%)	100 (18%)	25 (9%)
Alcohol	75 (6%)	19 (5%)	44 (8%)	12 (4%)
Marijuana	107 (9%)	37 (11%)	50 (9%)	20 (7%)
Any Illicit Drug Use	117 (10%)	41 (12%)	53 (10%)	23 (9%)
Infant characteristics				
Birth cohort				
2012-2015	592 (51%)	70 (20%)	378 (69%)	144 (54%)
2016-2019	404 (35%)	162 (47%)	149 (27%)	93 (35%)
2020-2023	164 (14%)	115 (33%)	19 (3%)	30 (11%)
Infant age at assessment (mean, SD)	1.1 (0.1)	1.1 (0.1)	1.1 (0.1)	1.1 (0.1)
Female sex	568 (49%)	159 (46%)	288 (53%)	121 (45%)
Preterm birth (< 37 weeks)	172 (15%)	51 (15%)	85 (16%)	36 (13%)
Received Early Intervention Services (EIS)	42 (4%)	16 (5%)	17 (3%)	9 (3%)

INSTI=integrase strand transfer inhibitor, NNRTI=non-nucleoside reverse transcriptase inhibitor, PI=protease inhibitor.

© 2025 Williams PL et al. *JAMA Network Open*.

eTable 4. Summary Statistics for Bayley-III and MCDI Scores, by Initial Maternal ARV Regimen

Characteristic	Total (N=1006)	Initial Maternal ARV Regimen in Pregnancy		
		INSTI-based regimen (N=306)	PI-based regimen (N=473)	NNRTI-based regimen (N=227)
Bayley-III Assessments				
Bayley-III composite scores (mean, SD)				
Cognitive	101.7 (14.2)	100.5 (13.9)	101.9 (13.6)	102.7 (15.6)
Language	95.5 (13.8)	94.9 (13.9)	95.8 (13.4)	95.6 (14.5)
Motor	96.4 (13.2)	95.2 (13.7)	96.8 (12.3)	97.1 (14.3)
Low Bayley Scores* (> 1.5 SDs below mean) (N, %)				
Cognitive	45 (4.5%)	12 (4.0%)	19 (4.1%)	14 (6.2%)
Language	84 (8.6%)	30 (10.1%)	35 (7.6%)	19 (8.5%)
Motor	56 (5.7%)	21 (7.0%)	21 (4.6%)	14 (6.2%)
Missing or invalid scores** (N, %)				
Cognitive	12	5	7	0
Language	24	9	12	3
Motor	23	8	13	2

	Total (N=1160)	Initial Maternal ARV Regimen in Pregnancy		
		INSTI-based regimen (N=347)	PI-based regimen (N=546)	NNRTI-based regimen (N=267)
MCDI Assessments				
MCDI Percentile scores (mean, SD)				
A-E total Gestures	47.8 (29.1)	47.3 (29.7)	48.2 (28.6)	47.5 (29.2)
Phrases Understood	57.1 (28.0)	56.2 (29.1)	57.2 (27.5)	57.9 (27.7)
Vocabulary Comprehension	48.4 (30.0)	50.4 (30.6)	47.1 (29.4)	48.3 (30.5)
Word Production	49.3 (22.6)	49.4 (22.5)	48.8 (22.3)	50.3 (23.5)
Low MCDI Scores (<10th percentile) (N, %)				
A-E total Gestures	188 (16.7%)	58 (17.1%)	83 (15.7%)	47 (17.9%)
Phrases Understood	85 (7.5%)	31 (9.1%)	37 (7.0%)	17 (6.5%)
Vocabulary Comprehension	158 (14.0%)	45 (13.3%)	70 (13.3%)	43 (16.4%)
Word Production	76 (6.7%)	19 (5.6%)	38 (7.2%)	19 (7.3%)
Missing or invalid scores* (N, %)				
(same for all domains)	32	8	19	5

*Based on the reference population with mean 100 and SD=15, 6.7% would be expected to have scores > 1.5 SDs below the mean (i.e., <77.5)

**Infants with missing or invalid scores are excluded from means and from the percentages with low scores.

© 2025 Williams PL et al. *JAMA Network Open*.

eTable 5. Sensitivity Analysis Results for Doubly Robust Estimates of Mean Differences in Bayley-III Scores by In Utero Exposure to Initial Maternal ART Regimen, Accounting for Correlated Outcomes Among Children Born to the Same Mother

	Bayley-III Outcome	INSTIs<br>Est mean (se)	PIs<br>Est mean (se)	NNRTIs<br>Est mean (se)	Adjusted mean difference* (95% CI)	P-value
Study Population: Overall						
		N=301	N=466	N=227		
**INSTIs vs NNRTIs**	Cognitive	101.60 (0.79)		104.09 (1.02)	-2.50 (-4.98, -0.01)	0.049
	Language	95.36 (1.74)		96.18 (1.01)	-0.83 (-4.72, 3.07)	0.68
	Motor	95.90 (0.79)		97.04 (1.25)	-1.14 (-4.00, 1.72)	0.43
**INSTIs vs PIs**	Cognitive	101.40 (1.03)	101.22 (0.66)		0.20 (-2.17, 2.57)	0.87
	Language	93.02 (2.51)	95.36 (0.67)		-2.32 (-7.36, 2.71)	0.37
	Motor	96.22 (1.02)	96.64 (0.58)		-0.41 (-2.69, 1.88)	0.73
Study Population: Infants born to mothers on ART at conception						
		N=167	N=216	N=142		
**INSTIs vs NNRTIs**	Cognitive	101.03 (1.08)		104.59 (1.97)	-3.52 (-7.91, 0.88)	0.12
	Language	95.00 (1.00)		96.65 (1.44)	-1.61 (-4.98, 1.77)	0.35
	Motor	96.32 (1.12)		97.37 (2.10)	-1.01 (-5.63, 3.62)	0.67
**INSTIs vs PIs**	Cognitive	101.59 (1.26)	103.22 (1.76)		-1.60 (-5.83, 2.63)	0.46
	Language	95.05 (1.16)	95.66 (1.09)		-0.57 (-3.55, 2.42)	0.71
	Motor	96.76 (1.34)	96.76 (1.45)		0.04 (-3.83, 3.91)	0.99
Study Population: Infants born to mothers who initiated ART during pregnancy						
		N=134	N=250	N=85		
**INSTIs vs NNRTIs**	Cognitive	101.92 (1.09)		105.59 (1.44)	-3.79 (-7.40, -0.19)	0.039
	Language	95.60 (1.21)		96.54 (1.74)	-1.00 (-5.16, 3.16)	0.64
	Motor	95.85 (1.11)		98.81 (1.58)	-3.05 (-6.85, 0.75)	0.11
**INSTIs vs PIs**	Cognitive	101.15 (1.47)	100.66 (0.91)		0.49 (-2.86, 3.84)	0.77
	Language	94.14 (1.48)	95.21 (0.83)		-1.07 (-4.30, 2.16)	0.51
	Motor	95.49 (1.27)	97.10 (0.81)		-1.61 (-4.57, 1.35)	0.29

**Average causal effect from doubly robust estimators, accounting for the following covariates in GEE linear regression adjustment and in inverse probability weighting for treatment regimen: maternal education, household income level, birth year (categorized as 2012-2015, 2016-2019, 2020-2023), maternal perinatal HIV status, geographic region, maternal age at delivery, maternal substance use in the first trimester (alcohol, tobacco, marijuana, each considered separately), and for regression adjustment only, sex at birth.*

GEE linear regression model fit using identity link and normal distribution, Inverse probability weights obtained by inverting predicted probabilities from GEE logistic regression model (logit link, binomial distribution).

© 2025 Williams PL et al. *JAMA Network Open*.

eTable 6. Sensitivity Analysis Results for Doubly Robust Estimates of Mean Differences in Bayley-III Scores by In Utero Exposure to Initial Maternal ART Regimen, Restricted to Infants of Mothers Who Continued the Same Regimen Type Throughout Pregnancy

	Bayley-III Outcome	INSTIs<br>Est mean (se)	PIs<br>Est mean (se)	NNRTIs<br>Est mean (se)	Adjusted mean difference* (95% CI)	P-value
Study Population: Overall						
		N=301	N=466	N=227		
**INSTIs vs NNRTIs**	Cognitive	100.98 (0.88)		103.70 (1.26)	-2.72 (-5.70, 0.26)	0.074
	Language	94.74 (0.84)		96.00 (1.22)	-1.26 (-4.12, 1.59)	0.39
	Motor	96.07 (0.78)		97.46 (1.34)	-1.39 (-4.39, 1.62)	0.37
**INSTIs vs PIs**	Cognitive	100.58 (1.21)	100.78 (0.84)		-0.20 (-3.07, 2.67)	0.89
	Language	95.04 (1.11)	96.05 (0.92)		-1.01 (-3.79, 1.77)	0.48
	Motor	96.19 (0.96)	97.11 (0.64)		-0.91 (-3.15, 1.32)	0.42
Study Population: Infants born to mothers on ART at conception						
		N=167	N=216	N=142		
**INSTIs vs NNRTIs**	Cognitive	100.75 (1.22)		104.81 (1.60)	-4.05 (-7.95, -0.15)	0.042
	Language	94.62 (1.23)		97.21 (1.78)	-2.59 (-6.73, 1.55)	0.22
	Motor	96.56 (1.26)		100.26 (1.62)	-3.70 (-7.75, 0.35)	0.073
**INSTIs vs PIs**	Cognitive	100.81 (1.26)	100.87 (1.90)		-0.06 (-4.48, 4.36)	0.98
	Language	95.94 (1.48)	97.63 (1.90)		-1.69 (-6.36, 2.98)	0.48
	Motor	96.74 (1.32)	96.90 (0.75)		-0.16 (-3.07, 2.75)	0.91
Study Population: Infants born to mothers who initiated ART during pregnancy						
		N=134	N=250	N=85		
**INSTIs vs NNRTIs**	Cognitive	100.89 (1.20)		106.58 (1.47)	-5.69 (-9.50, -1.89)	0.003
	Language	94.53 (1.12)		99.75 (2.00)	-5.22 (-9.72, -0.72)	0.023
	Motor	96.02 (1.01)		101.54 (1.92)	-5.53 (-9.78, -1.28)	0.011
**INSTIs vs PIs**	Cognitive	100.67 (1.76)	100.53 (1.12)		0.14 (-3.92, 4.21)	0.95
	Language	93.82 (1.48)	95.87 (1.04)		-2.04 (-5.51, 1.42)	0.25
	Motor	95.98 (1.21)	97.50 (1.05)		-1.52 (-4.66, 1.61)	0.34

**Average causal effect from doubly robust estimators, accounting for the following covariates in regression adjustment and in inverse probability weighting for treatment regimen: maternal education, household income level, birth year (categorized as 2012-2015, 2016-2019, 2020-2023), maternal perinatal HIV status, geographic region, maternal age at delivery, maternal substance use in the first trimester (alcohol, tobacco, marijuana, each considered separately), and for regression adjustment only, sex at birth.*

© 2025 Williams PL et al. *JAMA Network Open*.

eTable 7. Sensitivity Analysis Results for Doubly Robust Estimates of Mean Differences in Bayley-III Scores by In Utero Exposure to Initial Maternal ART Regimen, Further Adjusted for Maternal CD4 and VL During Pregnancy

	Bayley-III Outcome	INSTIs Est mean (se)	PIs Est mean (se)	NNRTIs Est mean (se)	Adjusted mean difference* (95% CI)	P-value
Study Population: Overall						
		N=301	N=466	N=227		
**INSTIs vs NNRTIs**	Cognitive	101.19 (0.85)		103.63 (1.12)	-2.45 (-5.19, 0.29)	0.080
	Language	94.54 (0.83)		96.44 (1.20)	-1.90 (-4.75, 0.95)	0.19
	Motor	95.87 (0.83)		96.99 (1.11)	-1.11 (-3.80, 1.58)	0.42
**INSTIs vs PIs**	Cognitive	101.31 (1.03)	100.66 (0.72)		0.66 (-1.78, 3.09)	0.60
	Language	95.05 (0.95)	95.12 (0.68)		-0.07 (-2.31, 2.17)	0.95
	Motor	95.82 (1.01)	96.35 (0.61)		-0.53 (-2.82, 1.76)	0.65
Study Population: Infants born to mothers on ART at conception						
		N=167	N=216	N=142		
**INSTIs vs NNRTIs**	Cognitive	100.83 (1.12)		103.42 (1.44)	-2.59 (-6.11, 0.93)	0.15
	Language	94.21 (1.14)		96.26 (1.46)	-2.04 (-5.64, 1.55)	0.26
	Motor	96.38 (1.22)		97.89 (1.29)	-1.52 (-4.95, 1.92)	0.39
**INSTIs vs PIs**	Cognitive	101.17 (1.16)	101.73 (1.03)		-0.55 (-3.53, 2.43)	0.72
	Language	95.01 (1.29)	95.57 (1.11)		-0.56 (-3.80, 2.68)	0.74
	Motor	96.00 (1.38)	95.78 (0.87)		0.22 (-2.92, 3.36)	0.89
Study Population: Infants born to mothers who initiated ART during pregnancy						
		N=134	N=250	N=85		
**INSTIs vs NNRTIs**	Cognitive	100.61 (1.26)		102.91 (2.18)	-2.30 (-7.27, 2.66)	0.36
	Language	94.16 (1.18)		95.86 (2.07)	-1.69 (-6.34, 2.95)	0.47
	Motor	95.04 (1.12)		97.15 (2.09)	-2.11 (-6.73, 2.52)	0.37
**INSTIs vs PIs**	Cognitive	100.62 (1.70)	99.94 (0.91)		0.68 (-3.08, 4.44)	0.72
	Language	93.30 (1.40)	94.85 (0.86)		-1.55 (-4.71, 1.61)	0.34
	Motor	94.74 (1.40)	96.77 (0.89)		-2.03 (-5.27, 1.21)	0.22

**Average causal effect from doubly robust estimators, accounting for the following covariates in regression adjustment and in inverse probability weighting for treatment regimen: maternal education, household income level, birth year (categorized as 2012-2015, 2016-2019, 2020-2023), maternal perinatal HIV status, geographic region, maternal age at delivery, maternal substance use in the first trimester (alcohol, tobacco, marijuana, each considered separately), maternal CD4 (earliest pregnancy CD4<250 cells/mL), maternal VL (earliest pregnancy VL<50 copies/mL), and for regression adjustment only, sex at birth.*

© 2025 Williams PL et al. *JAMA Network Open*.

eTable 8. Effect of Timing of ARV Initiation Within Drug Class on Bayley-III Outcomes, Unadjusted and Adjusted for Covariates

Initial Regimen Drug Class	Outcome	Timing of initiation	N	Estimated difference (95% CI)	P-value	Overall P-value[1]	Adjusted difference[2] (95% CI)	P-value (adj)	Overall P-value[1] (adj)
**INSTI**	Cognitive	Pre-conception	167	-2.13 (-4.52, 0.25)	0.080		-1.40 (-3.96, 1.16)	0.28	
		1st trimester	67	-4.06 (-7.60, -0.52)	0.025		-3.08 (-6.72, 0.56)	0.097	
		2nd/3rd trimester	67	1.69 (-1.86, 5.23)	0.35		1.59 (-1.97, 5.15)	0.38	
		Not initiated	693	(Ref)		0.030	(Ref)		0.18
	Language	Pre-conception	165	-0.66 (-3.00, 1.68)	0.58		-0.17 (-2.64, 2.30)	0.90	
		1st trimester	67	-3.69 (-7.15, -0.23)	0.037		-3.85 (-7.36, -0.34)	0.032	
		2nd/3rd trimester	65	1.64 (-1.87, 5.14)	0.36		0.88 (-2.59, 4.35)	0.62	
		Not initiated	685	(Ref)		0.12	(Ref)		0.15
	Motor	Pre-conception	165	-1.45 (-3.69, 0.79)	0.20		-0.73 (-3.13, 1.68)	0.55	
		1st trimester	67	-4.71 (-8.01, -1.40)	0.005		-3.96 (-7.36, -0.56)	0.023	
		2nd/3rd trimester	66	0.57 (-2.76, 3.89)	0.74		0.58 (-2.78, 3.93)	0.74	
		Not initiated	685	(Ref)		0.028	(Ref)		0.13
**NNRTI**	Cognitive	Pre-conception	142	1.28 (-1.26, 3.82)	0.32		1.43 (-1.10, 3.96)	0.27	
		1st trimester	42	1.59 (-2.81, 6.00)	0.48		0.99 (-3.36, 5.34)	0.66	
		2nd/3rd trimester	43	1.50 (-2.86, 5.86)	0.50		0.61 (-3.70, 4.92)	0.78	
		Not initiated	767	(Ref)		0.64	(Ref)		0.71
	Language	Pre-conception	141	0.27 (-2.21, 2.76)	0.83		1.25 (-1.19, 3.69)	0.32	
		1st trimester	41	-0.41 (-4.76, 3.93)	0.85		-0.64 (-4.88, 3.59)	0.77	
		2nd/3rd trimester	42	0.44 (-3.85, 4.74)	0.84		0.54 (-3.65, 4.73)	0.80	
		Not initiated	758	(Ref)		0.99	(Ref)		0.76
	Motor	Pre-conception	141	0.89 (-1.49, 3.26)	0.47		1.36 (-1.01, 3.73)	0.26	
		1st trimester	41	2.01 (-2.15, 6.17)	0.34		1.61 (-2.49, 5.72)	0.44	
		2nd/3rd trimester	43	-0.09 (-4.16, 3.97)	0.96		-1.11 (-5.13, 2.91)	0.59	
		Not initiated	758	(Ref)		0.72	(Ref)		0.54
**PI**	Cognitive	Pre-conception	215	1.03 (-1.22, 3.28)	0.37		0.47 (-1.84, 2.79)	0.69	
		1st trimester	125	-0.61 (-3.38, 2.15)	0.66		-1.09 (-3.91, 1.73)	0.45	
		2nd/3rd trimester	126	0.61 (-2.15, 3.37)	0.66		-0.24 (-3.10, 2.62)	0.87	
		Not initiated	528	(Ref)		0.72	(Ref)		0.80
	Language	Pre-conception	211	0.97 (-1.24, 3.18)	0.39		0.54 (-1.70, 2.77)	0.64	
		1st trimester	125	0.32 (-2.37, 3.02)	0.81		-0.41 (-3.12, 2.31)	0.77	
		2nd/3rd trimester	125	0.20 (-2.50, 2.89)	0.89		-0.70 (-3.46, 2.06)	0.62	
		Not initiated	521	(Ref)		0.86	(Ref)		0.86
	Motor	Pre-conception	212	0.13 (-1.97, 2.24)	0.90		-0.09 (-2.26, 2.08)	0.94	
		1st trimester	125	0.90 (-1.68, 3.48)	0.49		-0.06 (-2.70, 2.58)	0.97	
		2nd/3rd trimester	123	1.97 (-0.63, 4.56)	0.14		0.90 (-1.80, 3.60)	0.51	
		Not initiated	523	(Ref)		0.48	(Ref)		0.91

1 - P-value from 3 df AOVA test comparing all 4 groups.

2 - Adjusted model includes the following covariates: maternal education, household income level, birth year (categorized as 2012-2015, 2016-2019, 2020-2023), maternal perinatal HIV status, geographic region, maternal age at delivery, maternal substance use in the first trimester (alcohol, tobacco, marijuana, each considered separately), and sex at birth.

© 2025 Williams PL et al. *JAMA Network Open*.

eTable 9. Association of In Utero Exposure to Individual ARV Medications at Any Time During Gestation With Bayley-III Cognitive Scores Using a Hierarchical Model and a Full Model

			Hierarchical Model[1]		Full model[2]	
**Drug Class**	**ARV Drug**	**Exposed N (%)**	**Estimate (95% CI)**	**p-value**	**Estimate (95% CI)**	**p-value**
NRTIs	NRTIs (overall)	1005 (99.9%)	-0.11 (-1.65, 1.42)	0.89	-- N/A --	
	Lamivudine (3TC)	278 (27.6%)	-0.13 (-1.77, 1.51)	0.88	-0.68 (-5.67, 4.15)	0.79
	Abacavir (ABC)	132 (13.1%)	0.02 (-1.68, 1.72)	0.98	1.60 (-3.16, 6.21)	0.51
	Emtricitabine (FTC)	815 (81.0%)	-0.08 (-1.82, 1.67)	0.93	2.19 (-3.03, 7.25)	0.41
	Tenofovir DF (TDF)	677 (67.3%)	-0.34 (-2.06, 1.38)	0.70	-3.30 (-7.63, 0.90)	0.14
	Tenofovir AF (TAF)	192 (19.1%)	0.04 (-1.72, 1.79)	0.97	-0.83 (-5.40, 3.59)	0.72
	Zidovudine (ZDV)	204 (20.3%)	-0.18 (-1.85, 1.48)	0.83	0.70 (-3.05, 4.34)	0.71
NNRTIs	NNRTIs (overall)	257 (25.5%)	0.62 (-1.85, 3.08)	0.62	-- N/A --	
	Efavirenz (EFV)	41 (4.1%)	0.57 (-1.99, 3.13)	0.66	0.57 (-4.01, 5.01)	0.81
	Nevirapine (NVP)	16 (1.6%)	0.68 (-1.94, 3.31)	0.61	3.27 (-3.90, 10.21)	0.37
	Rilpivirine (RPV)	208 (20.7%)	0.59 (-1.78, 2.97)	0.62	0.64 (-2.09, 3.28)	0.65
PIs	PIs (overall)	529 (52.6%)	-0.40 (-2.60, 1.81)	0.73	-- N/A --	
	Atazanavir (ATV)	263 (26.1%)	-0.31 (-2.51, 1.89)	0.78	0.32 (-2.27, 2.82)	0.81
	Darunavir (DRV)	160 (15.9%)	-0.21 (-2.46, 2.03)	0.85	0.74 (-2.05, 3.44)	0.61
	Lopinavir/r (LPV/r)	124 (12.3%)	-0.66 (-2.99, 1.66)	0.58	-2.85 (-6.93, 1.09)	0.17
	Nelfinavir (NFV)	9 (0.9%)	-0.39 (-2.80, 2.02)	0.75	-1.04 (-11.17, 8.78)	0.84
INSTIs	INSTIs (overall)	389 (38.7%)	-0.72 (-2.84, 1.40)	0.51	-- N/A --	
	Elvitegravir (EVG)	134 (13.3%)	-0.59 (-2.78, 1.60)	0.60	0.04 (-3.11, 3.09)	0.98
	Dolutegravir (DTG)	152 (15.1%)	-0.78 (-2.98, 1.42)	0.49	-1.75 (-5.01, 1.41)	0.29
	Raltegravir (RAL)	106 (10.5%)	-0.73 (-2.92, 1.45)	0.51	-0.55 (-3.62, 2.43)	0.73
	Bictegravir (BIC)	45 (4.5%)	-0.77 (-3.06, 1.52)	0.51	-2.38 (-7.72, 2.79)	0.38

[1] Hierarchical linear model considers individual ARV drugs as random effects nested within drug class, and overall drug classes and other covariates as fixed effects.
[2] Full model includes individual ARV drugs and other covariates as fixed effects

Both sets of models adjust for maternal education, household income level, birth year (categorized as 2012-2015, 2016-2019, 2020-2023), maternal perinatal HIV status, geographic region, maternal age at delivery, maternal substance use in the first trimester (alcohol, tobacco, marijuana, each considered separately), and sex at birth.

© 2025 Williams PL et al. *JAMA Network Open*.

eTable 10. Association of In Utero Exposure to Individual ARV Medications at Any Time During Gestation With Bayley-III Language Scores Using a Hierarchical Model and a Full Model

			Hierarchical Model		Full model	
Drug Class	ARV Drug	Exposed N (%)	Estimate (95% CI)	p-value	Estimate (95% CI)	p-value
NRTIs	NRTIs (overall)	1005 (99.9%)	-0.88 (-2.37, 0.62)	0.25	-- N/A --	
	Lamivudine (3TC)	278 (27.6%)	-0.91 (-2.51, 0.70)	0.27	-2.01 (-6.87, 2.70)	0.42
	Abacavir (ABC)	132 (13.1%)	-0.74 (-2.41, 0.92)	0.38	1.42 (-3.18, 5.87)	0.55
	Emtricitabine (FTC)	815 (81.0%)	-0.82 (-2.53, 0.88)	0.34	0.70 (-4.33, 5.57)	0.79
	Tenofovir DF (TDF)	677 (67.3%)	-0.92 (-2.60, 0.76)	0.28	-1.59 (-5.78, 2.47)	0.46
	Tenofovir AF (TAF)	192 (19.1%)	-0.85 (-2.57, 0.87)	0.33	-1.95 (-6.36, 2.32)	0.39
	Zidovudine (ZDV)	204 (20.3%)	-1.01 (-2.63, 0.62)	0.22	-1.13 (-4.81, 2.44)	0.55
NNRTIs	NNRTIs (overall)	257 (25.5%)	-0.62 (-3.03, 1.79)	0.61	-- N/A --	
	Efavirenz (EFV)	41 (4.1%)	-0.71 (-3.23, 1.80)	0.58	-2.07 (-6.58, 2.30)	0.37
	Nevirapine (NVP)	16 (1.6%)	-0.52 (-3.10, 2.06)	0.69	4.39 (-2.73, 11.28)	0.23
	Rilpivirine (RPV)	208 (20.7%)	-0.63 (-2.95, 1.69)	0.59	-0.66 (-3.31, 1.90)	0.63
PIs	PIs (overall)	529 (52.6%)	-0.43 (-2.57, 1.71)	0.69	-- N/A --	
	Atazanavir (ATV)	263 (26.1%)	-0.65 (-2.78, 1.48)	0.55	-1.24 (-3.73, 1.17)	0.33
	Darunavir (DRV)	160 (15.9%)	-0.16 (-2.35, 2.02)	0.88	1.14 (-1.57, 3.75)	0.41
	Lopinavir/r (LPV/r)	124 (12.3%)	-0.54 (-2.80, 1.72)	0.64	0.28 (-3.66, 4.10)	0.89
	Nelfinavir (NFV)	9 (0.9%)	-0.37 (-2.72, 1.98)	0.76	5.65 (-3.61, 14.63)	0.23
INSTIs	INSTIs (overall)	389 (38.7%)	-1.02 (-3.08, 1.05)	0.33	-- N/A --	
	Elvitegravir (EVG)	134 (13.3%)	-0.85 (-2.99, 1.28)	0.43	0.08 (-2.95, 3.03)	0.96
	Dolutegravir (DTG)	152 (15.1%)	-0.93 (-3.08, 1.22)	0.40	-0.77 (-3.94, 2.31)	0.64
	Raltegravir (RAL)	106 (10.5%)	-1.27 (-3.40, 0.86)	0.24	-2.93 (-5.89, -0.06)	0.053
	Bictegravir (BIC)	45 (4.5%)	-1.01 (-3.26, 1.23)	0.38	-0.52 (-5.75, 4.55)	0.85

[1] Hierarchical linear model considers individual ARV drugs as random effects nested within drug class, and overall drug classes and other covariates as fixed effects.
[2] Full model includes individual ARV drugs and other covariates as fixed effects

Both sets of models adjust for maternal education, household income level, birth year (categorized as 2012-2015, 2016-2019, 2020-2023), maternal perinatal HIV status, geographic region, maternal age at delivery, maternal substance use in the first trimester (alcohol, tobacco, marijuana, each considered separately), and sex at birth.

© 2025 Williams PL et al. *JAMA Network Open*.

eTable 11. Association of In Utero Exposure to Individual ARV Medications at Any Time During Gestation With Bayley-III Motor Development Scores Using a Hierarchical Model and a Full Model

Drug Class	ARV Drug	Exposed N (%)	Hierarchical Model		Full model	
			Estimate (95% CI)	p-value	Estimate (95% CI)	p-value
NRTIs	NRTIs (overall)	1005 (99.9%)	-1.11 (-2.55, 0.33)	0.13	-- N/A --	
	Lamivudine (3TC)	278 (27.6%)	-1.17 (-2.73, 0.38)	0.14	0.42 (-4.26, 4.94)	0.86
	Abacavir (ABC)	132 (13.1%)	-1.10 (-2.72, 0.52)	0.18	-2.06 (-6.50, 2.24)	0.36
	Emtricitabine (FTC)	815 (81.0%)	-0.96 (-2.61, 0.70)	0.26	1.19 (-3.69, 5.91)	0.63
	Tenofovir DF (TDF)	677 (67.3%)	-1.02 (-2.64, 0.61)	0.22	-2.07 (-6.10, 1.84)	0.31
	Tenofovir AF (TAF)	192 (19.1%)	-1.16 (-2.83, 0.51)	0.17	-2.66 (-6.91, 1.47)	0.22
	Zidovudine (ZDV)	204 (20.3%)	-1.25 (-2.82, 0.33)	0.12	-1.92 (-5.45, 1.50)	0.29
NNRTIs	NNRTIs (overall)	257 (25.5%)	-1.06 (-3.38, 1.27)	0.37	-- N/A --	
	Efavirenz (EFV)	41 (4.1%)	-1.20 (-3.63, 1.23)	0.33	-3.74 (-8.05, 0.44)	0.090
	Nevirapine (NVP)	16 (1.6%)	-0.98 (-3.49, 1.52)	0.44	2.30 (-4.59, 8.98)	0.51
	Rilpivirine (RPV)	208 (20.7%)	-0.98 (-3.21, 1.25)	0.39	-0.57 (-3.12, 1.89)	0.66
PIs	PIs (overall)	529 (52.6%)	-1.58 (-3.64, 0.49)	0.13	-- N/A --	
	Atazanavir (ATV)	263 (26.1%)	-1.35 (-3.41, 0.70)	0.20	-0.66 (-3.06, 1.67)	0.59
	Darunavir (DRV)	160 (15.9%)	-1.64 (-3.74, 0.47)	0.13	-1.94 (-4.54, 0.57)	0.14
	Lopinavir/r (LPV/r)	124 (12.3%)	-1.79 (-3.98, 0.40)	0.11	-2.81 (-6.60, 0.87)	0.15
	Nelfinavir (NFV)	9 (0.9%)	-1.53 (-3.81, 0.75)	0.19	2.49 (-6.47, 11.18)	0.59
INSTIs	INSTIs (overall)	389 (38.7%)	-1.84 (-3.83, 0.14)	0.069	-- N/A --	
	Elvitegravir (EVG)	134 (13.3%)	-1.76 (-3.82, 0.30)	0.094	-1.15 (-4.09, 1.70)	0.44
	Dolutegravir (DTG)	152 (15.1%)	-1.77 (-3.84, 0.29)	0.093	-1.36 (-4.40, 1.59)	0.38
	Raltegravir (RAL)	106 (10.5%)	-1.98 (-4.03, 0.07)	0.059	-2.61 (-5.48, 0.17)	0.074
	Bictegravir (BIC)	45 (4.5%)	-1.86 (-4.03, 0.31)	0.092	-1.77 (-6.78, 3.09)	0.49

[1] Hierarchical linear model considers individual ARV drugs as random effects nested within drug class, and overall drug classes and other covariates as fixed effects.
[2] Full model includes individual ARV drugs and other covariates as fixed effects

Both sets of models adjust for maternal education, household income level, birth year (categorized as 2012-2015, 2016-2019, 2020-2023), maternal perinatal HIV status, geographic region, maternal age at delivery, maternal substance use in the first trimester (alcohol, tobacco, marijuana, each considered separately), and sex at birth.

© 2025 Williams PL et al. *JAMA Network Open*.

eFigure. Derivation of Study Population for MCDI Neurodevelopmental Analysis

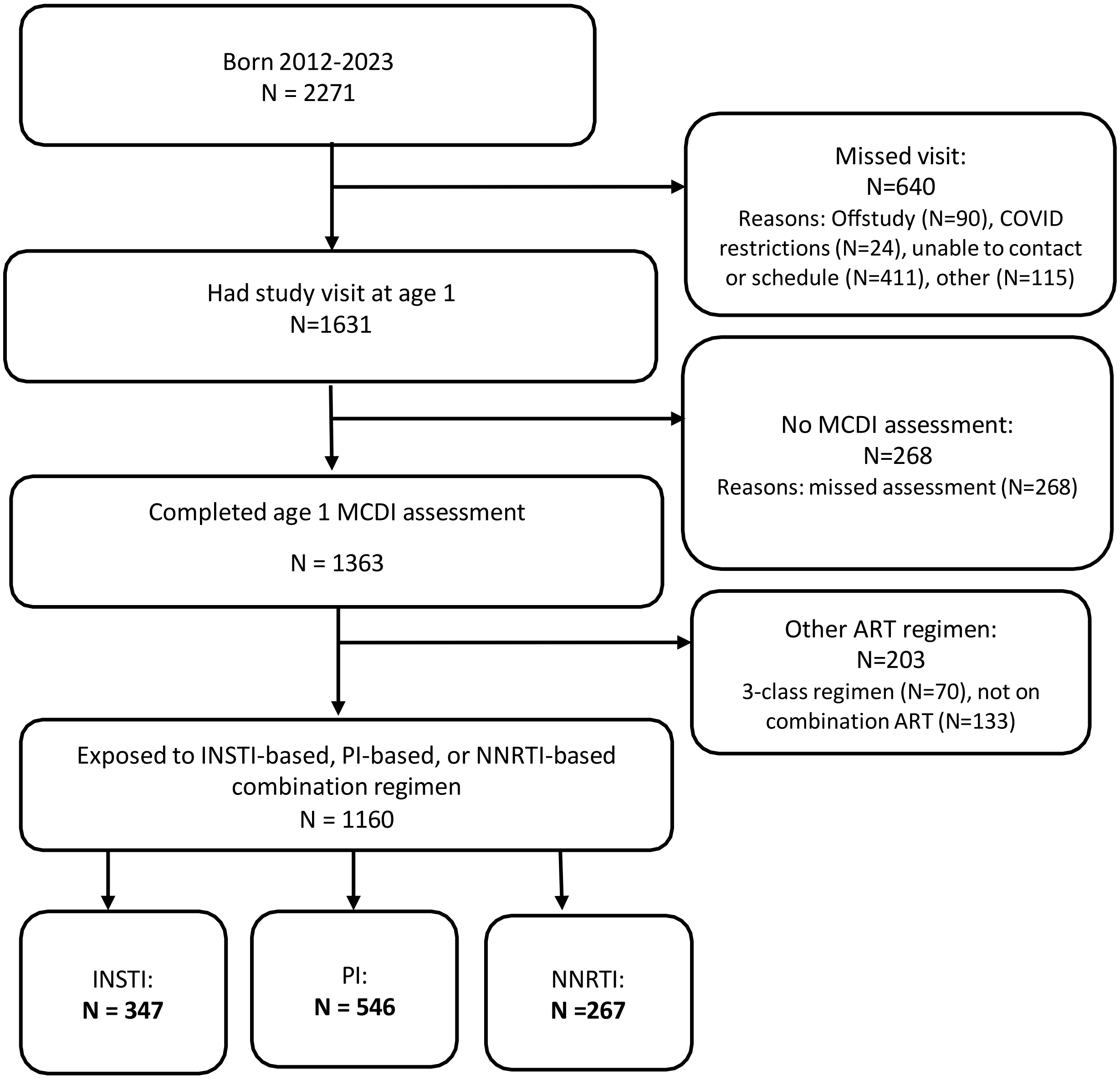

© 2025 Williams PL et al. *JAMA Network Open*.

eAppendix. Additional Acknowledgements

We thank the participants and families for their participation in PHACS, and the individuals and institutions involved in the conduct of the PHACS SMARTT study. The study was supported by the *Eunice Kennedy Shriver* National Institute of Child Health & Human Development (NICHD), Office of the Director, National Institutes of Health (OD), National Institute of Dental & Craniofacial Research (NIDCR), National Institute of Allergy and Infectious Diseases (NIAID), National Institute of Neurological Disorders and Stroke (NINDS), National Institute on Deafness and Other Communication Disorders (NIDCD), National Institute of Mental Health (NIMH), National Institute on Drug Abuse (NIDA), National Cancer Institute (NCI), National Institute on Alcohol Abuse and Alcoholism (NIAAA), and National Heart, Lung, and Blood Institute (NHLBI) through grants to the Harvard T.H. Chan School of Public Health (P01HD103133, Principal Investigators: Ellen Chadwick, Sonia Hernandez-Diaz, Jennifer Jao, Paige Williams; Program Director: Liz Salomon and HD052102: Principal Investigator: George R Seage III; Program Director: Liz Salomon) and with Tulane University School of Medicine (HD052104) (Principal Investigator: Russell Van Dyke; Co-Principal Investigator: Ellen Chadwick; Project Director: Patrick Davis). Data management services were provided by Frontier Science (Data Management Center Director: Suzanne Siminski), and regulatory services and logistical support were provided by Westat, Inc (Project Director: Tracy Wolbach).

The institutions and staff involved in the conduct of the PHACS SMARTT study can be found at https://phacsstudy.org/publications/smartt-acknowledgements/.

© 2025 Williams PL et al. *JAMA Network Open*.
