# Supplementary material for: Maternal Use of Integrase Strand Transfer Inhibitors During Pregnancy and Infant Neurodevelopment
Source: JAMA Netw Open. 2025 Nov 26;8(11):e2545652. doi: 10.1001/jamanetworkopen.2025.45652 (PMC12658668; doi:10.1001/jamanetworkopen.2025.45652)
Supplement: Supplement 2. — Data Sharing Statement [file jamanetwopen-e2545652-s002.pdf]

Data Sharing Statement

Williams. Maternal Use of Integrase Strand Transfer Inhibitors During Pregnancy and Infant Neurodevelopment. *JAMA Netw Open*. Published November 26, 2025.
doi:10.1001/jamanetworkopen.2025.45652

Data
Data available: Yes
Data types: Deidentified participant data
How to access data: https://dash.nichd.nih.gov/study/227210
When available: Beginning date: 01-01-2026

Supporting Documents
Document types: None

Additional Information
Who can access the data: Researchers must propose use of the data with a scientific proposal and obtain approval from the NICHD Data and Specimen Hub (DASH) team.
Types of analyses: For specific research proposals as approved by the NICHD DASH Team.
Mechanisms of data availability: After approval of proposal by DASH, a Data Use Agreement must be executed between the investigator's institution and DASH.
Any additional restrictions: Additional restrictions may be imposed by NICHD DASH as part of the DUA.
